# Which Variables Are Associated with the Magnitude of the Physical Fitness Response in Older Adults? An Analysis of Their Development and Influence

**DOI:** 10.3390/ijerph21081075

**Published:** 2024-08-16

**Authors:** Andressa Crystine da Silva Sobrinho, Larissa Chacon Finzeto, Mariana Luciano de Almeida, Guilherme da Silva Rodrigues, João Gabriel Ribeiro de Lima, Karine Pereira Rodrigues, Átila Alexandre Trapé, Lais Prado, Carlos Roberto Bueno Júnior

**Affiliations:** 1 Department of Graduate Studies in Clinical Medicine, Faculty of Medicine of Ribeirão Preto, University of São Paulo (FMRP-USP), Ribeirão Preto Campus, Avenida Bandeirantes 3900, Ribeirão Preto 14049-900, SP, Brazil; larissa.finzeto@usp.br (L.C.F.); ml.almeida@usp.br (M.L.d.A.); joao.ribeiro.lima@usp.br (J.G.R.d.L.); karodrigues@usp.br (K.P.R.);; 2 School of Physical Education and Sport of Ribeirão Preto, University of São Paulo (USP), Avenida Bandeirantes 3900, Ribeirão Preto 14049-900, SP, Brazil; atrape@usp.br (Á.A.T.); laispradonutricionista@gmail.com (L.P.)

**Keywords:** physical exercise, aging, training programs, personalized training, multiple linear regression, health, well-being

## Abstract

Regular physical exercise has proven to be an effective strategy for enhancing the health and well-being of older adults. However, there are still gaps in our understanding of the impacts of exercise on older adults with different health conditions, as well as in the customization of training programs according to individual capabilities. This study aimed to analyze the variables that influence the response of physical capabilities in older adults, considering their development over the aging process, with the goal of assisting professionals in creating personalized training programs. To achieve this, we conducted a cohort study involving 562 previously inactive adults and older adults who underwent anthropometric assessments, blood pressure measurements, and comprehensive physical tests. These assessments were conducted before and after a 14-week training program. Results indicated no significant variations in variables such as waist circumference (*p* = 0.0455, effect size = 0.10), body mass index (*p* = 0.0215, effect size = 0.15), systolic (*p* < 0.0001, effect size = 0.35) and diastolic blood pressure (*p* < 0.0001, effect size = 0.25) pre- and post-intervention. Strength tests, agility, the 6 min walk test (6MWT), and the back scratch test (BS) showed significant improvements post-intervention, with *p*-values all below 0.0001 and effect sizes ranging from 0.30 to 0.50. Multiple linear regression analyses revealed that lower initial values in physical capabilities were associated with more significant improvements during training (R^2^ = 0.73, *p* < 0.001). These results underscore that individualized guidance in training can lead to clinically meaningful improvements in physical performance and health among older adults, with effect sizes indicating moderate-to-large benefits (effect size range = 0.30 to 0.50). Therefore, personalized training programs are essential to maximize health benefits in this population.

## 1. Introduction

Aging is a natural process in the human life cycle, and with the increase in life expectancy, it has become an increasingly prevalent phenomenon in our society [1]. The aging process entails physiological, psychological, and social changes [1]. As individuals age, there are alterations in the musculoskeletal system, such as the loss of muscle mass, bone mineral density, and flexibility. Additionally, behavioral changes (e.g., decreased protein intake and reduced physical activity levels) contribute to a range of health issues, including cardiovascular diseases, osteoporosis, and obesity. These conditions affect the quality of life and independence of older adults [1,2]. In this context, it is important to emphasize that aging does not have to equate to frailty and limitations. Physical exercise can be an effective tool for improving the health and well-being of older adults [2].

Indeed, several studies have demonstrated that regular physical exercise can promote numerous health benefits for older adults, such as improved cardiorespiratory fitness, increased muscle strength, reduced risk of falls, and the control of blood pressure and glucose levels [2,3]. Older adults who engage in regular exercise exhibit greater autonomy and independence in performing activities of daily living [3].

To reap the advantages of physical exercise, it is important that the exercise program be customized to the unique requirements and capacities of each older individual, with due consideration to the physical constraints that may arise at this stage of life [3,4]. In this regard, it is essential to consider which physical capabilities should be optimized in old age, such as muscle strength, flexibility, motor coordination, balance, and aerobic capacity [1,4]. According to recent studies, a combination of resistance and aerobic exercises appears to be the most effective for improving physical capabilities in older adults [5].

However, despite the advancements in understanding the benefits of physical exercise in older adults [1], it is still necessary to comprehend the behaviors that regular physical exercise will exhibit as outcomes when comparing older adults with different health conditions.

In this context, it is important to consider which physical capabilities should be trained and to tailor the exercise program to the individual needs and capabilities of each older adult. However, there is still a scarcity of information on how variables should be manipulated to achieve the proposed exercise objectives.

The magnitude of the response to physical capabilities in older adults is an important consideration when prescribing physical exercises for this population. Several factors can influence the magnitude of the response, including age, prior physical activity level, the type and duration of training, as well as the individual’s health status and the order of training relative to their needs [6].

The aim of this article is to identify potential variables associated with the magnitude of the response to physical capabilities in older adults. Understanding these factors can assist healthcare professionals and physical education experts in designing personalized and tailored physical training programs that address the individual needs and limitations of older adults.

We hypothesize that individualized physical training programs tailored to the initial physical capabilities and health conditions of older adults will result in greater improvements in physical fitness and health markers compared to non-individualized programs.

## 2. Materials and Methods

### 2.1. Participants and Intervention

This study utilized data from 562 adults and older adults aged 50 to 70 who were insufficiently active (had not engaged in regular physical activity for at least 3 months), following the guidelines of the American College of Sports Medicine (ACSM). All data were collected by the Health, Genetics, and Physical Exercise Studies Center (NESGEF) of the School of Physical Education and Sport in Ribeirão Preto at the University of São Paulo (EEFERP-USP). This cohort database spans from 2016 to 2020 (the pre-pandemic period) and includes exercise interventions that lasted for 14 weeks.

**Figure 1:**
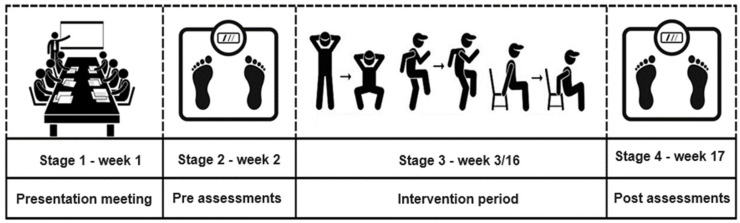
Experimental design.

Participants were recruited over the years through the Physical Education Program for Older Adults (PEFI), an outreach project of EEFERP-USP. The inclusion criteria for the study were as follows: age above 50 years, being a woman, enrollment in the PEFI project, and ability to participate in physical activities, i.e., no physical, visual, or cognitive limitations that would prevent the execution of the chosen tests. Additionally, participants had to be previously inactive, defined as not engaging in regular physical activity for at least 3 months according to the guidelines of the American College of Sports Medicine (ACSM). The exclusion criteria included the presence of any medical condition that contraindicated participation in physical exercise, as assessed by a healthcare professional; limitations that would impair the ability to safely perform the physical assessments or follow the training protocol, such as severe arthritis, uncontrolled hypertension, or advanced cardiovascular disease; participants who did not provide informed consent; individuals who were already participating in another structured physical activity program; and participants who had more than 25% absences during the intervention period. Assessments were conducted one week before and one week after 14 weeks of the physical training program (Figure 1).

The interventions were conducted 2 to 3 times per week, totaling 180 min of exercise per week. The training sessions were separated by 1 day of rest per week. Of these 180 min, 60 min were dedicated to aerobic exercises, such as outdoor walking and treadmill use, while the remaining 120 were devoted to strength training. Upper and lower extremity exercises were performed in the same strength training session. This training followed the multicomponent method and included exercises performed with dumbbells, resistance bands, kettlebells, and weight machines [3,5,7].

All participants exercised together in groups of 20 individuals, and necessary adjustments were made for any participant who had limitations with the proposed exercises to ensure safety. All sessions were planned and conducted by a team of 3 to 5 physical education professionals.

Throughout the training session, participants were asked to report their perceived exertion index at the end of each session using the adapted Borg scale [8]. At the beginning of the session, participants were asked to maintain an effort in the range of 5 to 8 on the above scale to ensure that the training was performed at an appropriate intensity.

### 2.2. Ethical Considerations

The interventions were periodically approved by the Research Ethics Committee of the School of Physical Education and Sport in Ribeirão Preto at the University of São Paulo (EEFERP-USP, CAAE 45889915.0.0000.5659, CAAE: 79582817.0.0000.5659, CAAE: 45889915.0.0000.5659). The details of the interventions are registered on the Brazilian Clinical Trials Registry Platform (ReBEC: Protocol Number: RBR-3g38dx).

### 2.3. Assessments

The following parameters were assessed according to the literature [7]: height, body mass (kg), and body mass index (BMI) (kg/m^2^). The waist circumference (WC) was measured at the midpoint between the last rib and the iliac crest, with the individual in a standing position and at the maximum point of normal expiration [7]. The hip circumference, using a measuring tape, was measured at the level of the largest circumference of the buttocks, without folds, and aligned horizontally. The tape had an accuracy of 1 mm. An automatic blood pressure monitor (OMRON HEM-7113, Omron Healthcare, Kyoto, Japan) was employed to assess blood pressure (in mmHg). Blood pressure measurements were taken in triplicate, with the participant at rest for a minimum of five minutes, following the guidelines outlined in the Brazilian Guideline for Arterial Hypertension [9].

Blood pressure, along with anthropometric data, was used in the study only for the characterization of the sample. In addition, these measurements were used to illustrate the differences in these variables before and after the intervention, thus identifying which variables showed improvements after the intervention.

### 2.4. Physical Test—Physical Capabilities

#### 2.4.1. Thirty-Second (30 s) Chair Stand Test

The 30 s Chair Stand (CS), validated by Rikli and Jones (1999), was conducted to assess the muscular strength of the lower limbs. This test quantifies the number of times an individual can, as quickly as possible, sit and stand from a chair in 30 s. The participant began the test seated in the middle of a chair seat (43 cm in height) with an upright posture, feet on the ground, and arms crossed over the chest. The evaluator instructed the participants to perform the maximum number of repetitions (sitting and standing from the chair) in 30 s [10].

#### 2.4.2. Elbow Flexion and Extension

In the elbow flexion and extension test (EFE), participants performed the repetitive movement of flexing and extending their dominant arm’s elbow with a 2.27 kg dumbbell as many times as possible within a 30 s timeframe.

#### 2.4.3. Hand Grip Strength

The hand grip strength test (HGS) followed the position approved by the American Society of Hand Therapists (ASHT), which is considered the “gold standard” for conducting the test. Participants were comfortably seated with the shoulder slightly adducted, the elbow flexed at 90 degrees, the forearm in a neutral position, and the wrist’s position ranging from 0° to 30° of extension [11].

#### 2.4.4. Cardiorespiratory Capacity

To assess cardiorespiratory capacity, the six-minute walk (6MWT) test was conducted. The distance covered was recorded during this test. The course was a rectangle measuring 4.57 by 18.28 m, and participants walked as close as possible to the cones at the highest possible speed for six minutes without running [10].

#### 2.4.5. Flexibility

Flexibility was evaluated using two tests proposed by Rikli and Jones (1999): Back Scratch (BS), which measures the distance between the distal ends of the two middle fingers of the hands using a ruler, and Chair Sit and Reach Test (SAR), which employs a chair and involves measuring the distance between the distal end of the middle finger of the hand and the distal end of the third toe [10].

#### 2.4.6. Agility

The AGIL test, developed by Osness in 1990, is a valuable tool for assessing an individual’s agility. This test measures functional mobility and lower limb performance by recording the time it takes for a person to navigate a course. The course consists of a chair placed within a marked area between two cones, positioned 1.50 m behind the starting point and 1.80 m to each side. The time recorded in this test provides valuable insights into an individual’s agility and dynamic balance capabilities [12].

#### 2.4.7. Sample Size and Statistical Power

The current study included a sample size of 562 previously inactive adults and older adults, which is a substantial cohort for this type of research. However, to enhance the robustness and generalizability of our findings, it is crucial to discuss the statistical power of the study.

#### 2.4.8. Sample Size Calculation

To ensure that our study was adequately powered to detect significant effects, a priori power analysis was conducted using G*Power 3.1 software (Heinrich-Heine-Universität Düsseldorf, Düsseldorf, Germany). The analysis was based on the following parameters: Effect Size (f^2^): 0.15 (medium effect size); Alpha Level (α): 0.05; Power (1-β): 0.80; Number of Predictors: 7.

Using these parameters, the calculated minimum sample size required to detect a statistically significant effect was 103 participants. Our study’s sample size of 562 participants exceeds this requirement, providing a robust basis for detecting even small to moderate effect sizes. This substantial sample size enhances the reliability and validity of our findings, ensuring that the study is adequately powered to detect the intended effects.

#### 2.4.9. Statistical Analysis

The statistical analyses were conducted following the intention-to-treat principle, which considers all participants according to their original allocation, regardless of treatment adherence or study completion. The collected data were organized into a two-way database using Microsoft Excel, version 2013 (Microsoft Corporation, Redmond, WA, USA). The data are presented as the mean and standard deviation. The Kolmogorov–Smirnov test was employed to assess data normality, and variances were examined using the Levene test. The impact of the training was analyzed using the independent sample *t*-test, a statistical method for comparing two independent groups.

Multiple linear regression analysis was conducted using the relative delta (Δ) of the studied variables. The delta variation (Δ = (Post-training − Pre-training)/Pre-training) between the pre- and post-intervention assessments was employed to quantify changes in quantitative variables. Multiple linear regression was employed to analyze the interactions between physical capabilities and their magnitude of response, adjusted for age. The “Hierarchical” entry method was utilized in the multiple linear regression model, which involves inserting variables into the model based on their importance, following a set of predefined criteria. Adjusted estimates (β) with a 95% confidence interval (CI) were calculated for all variables in the model. The adjusted R-squared (R^2^) was examined to determine the percentage of variation coefficient determination. Additionally, the standard error (SE) and the t-value (t) were calculated for all variables in the model to assess the statistical significance of the results.

The multiple linear regression analysis was conducted using the Δ (delta) of the variables as dependent variables and the Δ and pre-intervention values as predictors. Data were analyzed using the Statistical Package for the Social Sciences (SPSS), version 20.0 (IBM Corporation, Armonk, NY, USA). The results are presented as the mean (standard deviation, SD).

## 3. Results

In total, the data of 562 individual women were analyzed. There was no evidence of differences in the mean ages of the groups that underwent physical training during different periods from 2016 to 2020. Notably, variables including waist circumference, body mass index, systolic and diastolic blood pressure, strength tests, agility, 6MWT, and BS performance showed evidence of improvement following the physical training intervention (Table 1).

### 3.1. Physical Capability—Strength

Analyzing the overall strength models in the ‘elbow flexion and extension’ variable, we observed that the variables with better performance in the pre-moment were CS (R^2^ = 0.70), BS performance (R^2^ = 0.73), and EFE (R^2^ = 0.67). These variables influenced the magnitude of the response in the EFE test. It is worth noting that a negative value in the pre-intervention variable for EFE (R^2^ = 0.67) indicates that individuals who began with lower values exhibited a greater response magnitude to the physical training (Table 2).

When examining the influence by response magnitude, it is observed that the magnitude of performance (delta) of the physical capabilities such as CS (R^2^ = 0.54), 6MWT (R^2^ = 0.61), RHGS (R^2^ = 0.71), and LHGS (R^2^ = 0.72) contributed to amplifying the response magnitude of the EFE variable (Table 2).

Residual Analysis: The Durbin–Watson test (2.036) revealed no evidence of autocorrelation in the residuals (*p* > 0.05), confirming the robustness of the model.

Multicollinearity Assessment: Multicollinearity was thoroughly examined, with detailed results provided in Table 2. Tolerance values and VIFs indicate the absence of multicollinearity issues in this model.

Model Significance: The multiple linear regression model demonstrated statistical significance (F (7, 566) = 215.00, *p* < 0.001), with a coefficient of determination (R^2^) of 0.73 and an adjusted R^2^ of 0.712. These findings suggest that the model can account for 72.7% of the variance in the dependent variable, adjusted for the number of predictors.

Regression Equation: ΔEFE = 0.478 + 0.395 × (ΔSST) + 0.25 × (ΔGMWT) + 0.04 × (Pre EFE); + 0.02 × (Pre SST) + 0.18 × (ΔLHGS) + 0.16 × (ΔRHGS) + 0.004 × (Pre RBB).

The interpretation of the first variable in Table 2 is as follows: The estimated coefficient of the variable DELTA CS is 0.46, which means that a one-unit increase in the variable DELTA EFE is associated with a 0.46-unit increase in the dependent variable DELTA EFE. The variable DELTA CS has a significant impact on the dependent variable, DELTA EFE, explaining 54% of the variation. The estimated coefficient of the variable DELTA CS is 0.46 (*p* < 0.001; t = 1.35; 95% CI = 0.34 to 0.48).

The t-value for the estimated coefficient of the variable DELTA CS is 1.35 (*p* < 0.05). This means that the estimated coefficient is significant at the 0.05 significance level. The standard error of the estimated coefficient is 0.34, indicating that the estimated coefficient is estimated with a precision of 34%. The R^2^ for the variable DELTA CS is 0.54, signifying that the variable DELTA CS explains 54% of the variation in the dependent variable. The confidence interval for the estimated coefficient of the variable DELTA CS is 0.34 to 0.48, meaning that, with 95% confidence, the true value of the estimated coefficient falls within this interval.

In Table 3, for the CS variable, we see that the variables with better performance at the pre-moment, such as SAR (R^2^ = 0.64) and EFE (R^2^ = 0.62), influenced the magnitude of response of the variable, potentially suggesting these as fundamental capabilities that assist in the initial development of it. When we observe the negative value in the pre-variable for BS (R^2^ = 0.63) and CS (R^2^ = 0.60), we notice that individuals who started with lower values had a better chance of developing during the training.

When examining the influence of response magnitude, it is observed that the magnitude of performance (delta) in physical capabilities such as DELTA EFE (R^2^ = 0.54), DELTA 6MWT (R^2^ = 0.57), and DELTA LHGS (R^2^ = 0.63) contributed to amplifying the response magnitude of the CS variable (Table 3).

The interpretation of the first variable in Table 3 is as follows: The t-value for the estimated coefficient of the variable DELTA EFE is 8.86 (*p* < 0.05). This indicates that the estimated coefficient is significant at the 0.05 significance level. The standard error of the estimated coefficient is 0.07, meaning that the coefficient is estimated with a precision of 7%.

The R^2^ for the variable DELTA EFE is 0.54, which implies that this variable explains 54% of the variation in the dependent variable. The confidence interval for the estimated coefficient of the variable DELTA EFE is 0.61 to 0.81, suggesting that, with 95% confidence, the true value of the estimated coefficient falls within this interval.

In Table 3, upon examining the residuals using the Durbin–Watson test (1.542), no indications of autocorrelation were found within the residuals (*p* > 0.05), underscoring the reliability of the model. The presence of multicollinearity was meticulously investigated, and a comprehensive overview of the results is outlined in Table 3. Examination of tolerance values and VIFs revealed no discernible issues pertaining to multicollinearity within this model.

The multiple linear regression model exhibited statistical significance (F (7, 566) = 143.52, *p* < 0.001), featuring a coefficient of determination (R^2^) of 0.640 and an adjusted R^2^ of 0.635. These results imply that the model can explain a considerable percentage of the variance in the dependent variable, adjusting for the number of predictors employed.

The regression equation derived from the analysis is as follows: ΔSST = 0.145 + 0.62 × (ΔEFE) + 0.15 × (Δ6MWT) + (−0.24 × (Pre SST)) + 0.18 × (Pre EFE) + 0.10 × (ΔLHGS) + (−0.08 × (Pre RBB)) + 0.06 × (Pre SAR)

In Table 4, for the right-hand grip strength (RHGS) variable, we observe that variables with better performance at the pre-moment, such as 6MWT (R^2^ = 0.41), agility (R^2^ = 0.41), and EFE (R^2^ = 0.38), influenced the magnitude of the variable’s response, potentially suggesting these as fundamental capabilities that assist in the initial development of it. When considering the negative value in the pre-intervention variable for EFE (R^2^ = 0.67), it is evident that individuals starting with lower values had a better chance of improvement during training. Similarly, when examining the negative value in the pre-intervention variable for BS (R^2^ = 0.39), it is apparent that individuals starting with lower values had a better chance of improvement during training.

When examining the influence by response magnitude, it is observed that the magnitude of performance (delta) in physical capabilities such as LHGS 30% (R^2^ = 0.30), DELTA 6MWT 35% (R^2^ = 0.35), DELTA AGILITY 36% (R^2^ = 0.36), and DELTA EFE 37% (R^2^ = 0.37) contributed to amplifying the response magnitude of the RHGS variable in Table 4.

The interpretation of the first variable in Table 4 is as follows: The estimated coefficient for the variable DELTA LHGS is 0.19, which means that a 1-unit increase in the variable DELTA LHG is associated with a 0.19-unit increase in the dependent variable, DELTA RHGS. The variable DELTA LHGS has a significant impact on the dependent variable, DELTA LHGS, explaining 30% of the variation. The estimated coefficient for the variable DELTA LHGS is 0.19 (*p* < 0.001; t = 3.80; 95% CI = 0.17 to 0.21).

Residual Analysis: The Durbin–Watson test (1.504) revealed no evidence of autocorrelation in the residuals (*p* > 0.05), confirming the robustness of the model. Multicollinearity Assessment: Multicollinearity was thoroughly examined, with detailed results provided in Table 4. Tolerance values and VIFs indicate the absence of multicollinearity issues in this model.

Model Significance: The multiple linear regression model demonstrated statistical significance (F (8, 565) = 51.558, *p* < 0.001), with a coefficient of determination (R^2^) of 0.41 and an adjusted R^2^ of 0.40. These findings indicate that the model explains 41% of the variance in the dependent variable after adjusting for the number of predictors.

Regression Equation: ΔRHGS = 0.383 + 0.19 × (ΔLHGS) + 0.17 × (Δ6MWT) + 0.14 × (ΔAGILITY); + 0.21 × (ΔEFE) + 0.11 × (Pre EFE) + (−0.10 × (Pre RBB)) + 0.11 × (Pre 6MWT) + 0.08 × (Pre AGILITY)

In Table 5, regarding the LHGS variable, we can observe that variables with better performance in the pre-moment, such as LHGS (R^2^ = 0.59), 6MWT (R^2^ = 0.60), and AGILITY (R^2^ = 0.61), influenced the magnitude of the response variable. This suggests that these variables may represent fundamental abilities that aid in initial development. When we observe the negative value in the pre-RHGS variable (R^2^ = 0.58), it indicates that individuals who started with lower values had a better chance of improvement during the training.

When examining the influence of response magnitude, it is observed that the magnitude of performance (DELTA) in physical capabilities such as DELTA RHGS (R^2^ = 0.56), DELTA 6MWT (R^2^ = 0.53), DELTA AGILITY (R^2^ = 0.57), DELTA EFE (R^2^ = 0.53), and DELTA CS (R^2^ = 0.60) contributed to amplifying the response magnitude of the LHGS variable in Table 5.

The interpretation of the first variable in Table 5 is as follows: The t-value of the estimated coefficient for the variable DELTA 6MWT is 3.30 (*p* < 0.05). This means that the estimated coefficient is significant at the 0.05 significance level. The standard error of the estimated coefficient is 0.10. This means that the estimated coefficient is estimated with a precision of 10%. The R^2^ for the variable DELTA 6MWT is 0.47. This means that the DELTA 6MWT variable explains 47% of the variation in the dependent variable. The confidence interval for the estimated coefficient of the DELTA 6MWT variable is from 0.24 to 0.39. This means that, with 95% confidence, the true value of the estimated coefficient is within this interval.

In Table 5, upon examining the residuals using the Durbin–Watson test (1.785), no indications of autocorrelation were found within the residuals (*p* > 0.05), underscoring the reliability of the model. The presence of multicollinearity was meticulously investigated, and a comprehensive overview of the results is outlined in Table 5. Examination of tolerance values and VIFs revealed no discernible issues pertaining to multicollinearity within this model.

The multiple linear regression model exhibited statistical significance (F (9, 564) = 87.048, *p* < 0.001), featuring a coefficient of determination (R^2^) of 0.610 and an adjusted R^2^ of 0.603. These results imply that the model can explain a considerable percentage of the variance in the dependent variable, adjusting for the number of predictors employed.

The regression equation derived from the analysis is as follows: ΔLHGS = 0.527 + 0.33 × (Δ6MWT) + 0.16 × (ΔEFE) + 0.13 × (ΔRHGS) + 0.20 × (ΔAGILITY) + (−0.23 × (Pre RHGS)) + 0.14 × (Pre LHGS) + 0.10 × (ΔSST) + 0.09 × (Pre 6MWT) + 0.07 × (Pre AGILITY)

### 3.2. Flexibility Capacity

In Table 6 and Table 7, for the flexibility variables, when considering the influence on the magnitude of response, we observe that the performance level of the physical abilities, DELTA SAR 8% (R^2^ = 0.08) and DELTA BS 8% (R^2^ = 0.08), contributed to the amplification of the response magnitude in an inverse manner with respect to each other.

The estimated coefficient for the variable DELTA SAR is 0.29, which means that an increase of 1 unit in the DELTA SAR variable is associated with an increase of 0.29 units in the dependent variable, DELTA BS. The DELTA SAR variable has a significant impact on the dependent variable, DELTA BS, explaining 8% of the variation. The estimated coefficient for the variable DELTA SAR is 0.29 (*p* < 0.001; t = 5.80; 95% CI = 0.18 to 0.32).

The value of the t-statistic for the estimated coefficient of the DELTA SAR variable is 5.80 (*p* < 0.05). This indicates that the estimated coefficient is significant at the 0.05 significance level. The standard error of the estimated coefficient is 0.05, which means that the estimated coefficient is estimated with a precision of 5%. The R^2^ for the DELTA SAR variable is 0.08, indicating that it explains 8% of the variation in the dependent variable. The confidence interval for the estimated coefficient of the DELTA SAR variable is 0.18 to 0.32, meaning that, with 95% confidence, the true value of the estimated coefficient falls within this interval.

The estimated coefficient for the variable DELTA BS is 0.29, which means that an increase of 1 unit in the DELTA BS variable is associated with an increase of 0.29 units in the dependent variable, DELTA SAR. The DELTA BS variable has a significant impact on the dependent variable, DELTA SAR, explaining 8% of the variation. The estimated coefficient for the variable DELTA BS is 0.29 (*p* < 0.001; t = 5.80; 95% CI = 0.18 to 0.32).

The value of the t-statistic for the estimated coefficient of the DELTA BS variable is 5.80 (*p* < 0.05). This indicates that the estimated coefficient is significant at the 0.05 significance level. The standard error of the estimated coefficient is 0.05, which means that the estimated coefficient is estimated with a precision of 5%. The R^2^ for the DELTA BS variable is 0.08, indicating that it explains 8% of the variation in the dependent variable. The confidence interval for the estimated coefficient of the DELTA BS variable is 0.18 to 0.32, meaning that, with 95% confidence, the true value of the estimated coefficient falls within this interval.

In Table 6 and Table 7, upon examining the residuals using the Durbin–Watson test (1.941 and 1.902, respectively), no indications of autocorrelation were found within the residuals (*p* > 0.05), underscoring the reliability of the model. The presence of multicollinearity was meticulously investigated, and a comprehensive overview of the results is outlined in Table 6 and Table 7. Examination of tolerance values and VIFs revealed no discernible issues pertaining to multicollinearity within this model.

The regression equation derived from the analysis is as follows:

ΔRBB = 0.403 + 0.29 × (ΔSAR)(1)

ΔSAR = 0.116 + 0.29 × (ΔRBB)(2)

### 3.3. Cardiorespiratory Capacity

In Table 8, analyzing the general models of cardiorespiratory capacity for the walking variable, we can observe that the variables with better performance in the pre-moment, such as CS (R^2^ = 0.65), RHGS (R^2^ = 0.62), EFE (R^2^ = 0.61), and AGILITY (R^2^ = 0.64), influenced the magnitude of the response of the variable. This suggests that these may be fundamental capacities that assist in the development of this capacity. When we observe the negative value in the pre-age variable (R^2^ = 0.62), it indicates that individuals who started with lower values had a better chance of improvement during the training.

When we examine the influence by magnitude of responses, we observe that the performance levels of the physical abilities, including DELTA LHGS (R^2^ = 0.47), DELTA EFE (R^2^ = 0.56), DELTA AGILITY (R^2^ = 0.59), DELTA RHGS (R^2^ = 0.63), and DELTA CS (R^2^ = 0.64), contributed to the amplification of the response magnitude of the 6MWT variable in Table 8.

The regression equation derived from the analysis is as follows: Δ6MWT = 0.693 + 0.28 × (ΔLHGS) + 0.24 × (ΔEFE) + 0.19 × (ΔAGILITY) + 0.08 × (Pre EFE) + (−0.09 × (AGE)) + 0.10 × (Pre RHGS) + 0.10 × (ΔRHGS) + 0.15 × (ΔSST) + 0.07 × (Pre AGILITY) + 0.07 × (Pre SST)

The interpretation of the first variable in Table 8 is as follows: The t-value for the estimated coefficient of the variable DELTA LHGS is 2.15 (*p* < 0.05). This means that the estimated coefficient is statistically significant at the 0.05 significance level. The standard error of the estimated coefficient is 0.13, indicating that the estimated coefficient is precise to within 13%. The R^2^ for the variable DELTA LHGS is 0.47, which means that it explains 47% of the variance in the dependent variable. The confidence interval for the estimated coefficient of the variable DELTA LHGS is from 0.22 to 0.57. This means that, with 95% confidence, the true value of the estimated coefficient falls within this interval.

In Table 8, upon examining the residuals using the Durbin–Watson test (1.065), no indications of autocorrelation were found within the residuals (*p* > 0.05), underscoring the reliability of the model. The presence of multicollinearity was meticulously investigated, and a comprehensive overview of the results is outlined in Table 8. Examination of tolerance values and VIFs revealed no discernible issues pertaining to multicollinearity within this model.

The multiple linear regression model exhibited statistical significance (F (10, 563) = 112.194, *p* < 0.001), featuring a coefficient of determination (R^2^) of 0.65 and an adjusted R^2^ of 0.63. These results imply that the model can explain a considerable percentage of the variance in the dependent variable, adjusting for the number of predictors employed.

The regression equation derived from the analysis is as follows: Δ6MWT = 0.693 + 0.28 × (ΔLHGS) + 0.24 × (ΔEFE) + 0.19 × (ΔAGILITY) + 0.08 × (Pre EFE) + (−0.09 × (AGE)) + 0.10 × (Pre RHGS) + 0.10 × (ΔRHGS) + 0.15 × (ΔSST) + 0.07 × (Pre AGILITY) + 0.07 × (Pre SST)

In Table 9, when we observe the negative values in the variables PRE for CS (R^2^ = 0.50), AGILITY (R^2^ = 0.41), and 6MWT (R^2^ = 0.49), we notice that individuals who started with lower values had a better chance of improvement during the training. Examining the influence based on response magnitude, we observe that the performance improvements in physical capacities such as DELTA 6MWT (35%, R^2^ = 0.35), DELTA EFE (48%, R^2^ = 0.48), RHGS (50%, R^2^ = 0.50), and DELTA LHGS (47%, R^2^ = 0.47) significantly contributed to the increase in the response magnitude of the AGILITY variable, as shown in Table 9.

The interpretation of the first variable in Table 9 is as follows: The estimated coefficient for the variable DELTA 6MWT is 0.28, which means that a 1-unit increase in the DELTA 6MWT variable is associated with a 0.28-unit increase in the dependent variable, DELTA AGILITY. The DELTA 6MWT variable has a significant impact on the dependent variable, DELTA AGILITY, explaining 54% of the variation. The estimated coefficient for the DELTA 6MWT variable is 0.28 (*p* < 0.001; t = 2.15; 95% CI = 0.19 to 0.37).

Residual Analysis: The Durbin–Watson test (1.813) revealed no evidence of autocorrelation in the residuals (*p* > 0.05), confirming the robustness of the model. Multicollinearity Assessment: Multicollinearity was thoroughly examined, with detailed results provided in Table 9. Tolerance values and VIFs indicate the absence of multicollinearity issues in this model.

Model Significance: The multiple linear regression model demonstrated statistical significance (F (7, 566) = 83.897, *p* < 0.001), with a coefficient of determination (R^2^) of 0.50 and an adjusted R^2^ of 0.498. These findings suggest that the model can account for 50% of the variance in the dependent variable, adjusted for the number of predictors.

Regression Equation: ΔAGILITY = 1.28 + 0.28 × (Δ6MWT) + (−0.33 × (Pre AGILITY)) + 0.23 × (ΔLHGS); + 0.14 × (ΔEFE) + (−0.10 × (Pre 6MWT)) + 0.13 × (Δ RHGS) + (−0.07 × (Pre SST)

## 4. Discussion

In our results, we observed an improvement in anthropometric parameters and the studied physical capacities. The present study shows that the applied intervention was important in improving physical fitness and health markers, demonstrating the effectiveness of targeted physical training programs. Specifically, the exercise program not only improved all physical abilities but also achieved reductions in blood pressure, with reductions greater than 5 mmHg, which are known to significantly reduce patient morbidity [3,5,7]. In addition, the program improved physical abilities such as increased muscle strength, agility, and aerobic endurance, thereby reducing the risk of loss of physical independence. These improvements highlight the importance of comprehensive and targeted exercise interventions for the elderly population to promote overall health and functional independence [5,7].

The results of this study are robust, supported by a power analysis that confirms the study’s capability to detect small to moderate effect sizes. This robustness is evidenced by the statistically significant improvements observed in various physical capabilities post-intervention. These improvements highlight the importance of comprehensive and targeted exercise interventions for this population to promote overall health and functional independence.

The multiple linear regression models indicate that participants’ initial physical inactivity status at the beginning of the training program significantly affected their subsequent improvements. Ignasiak et al. (2017) [13] highlight the importance of maintaining functional capacities such as muscular strength, agility, and aerobic endurance to prevent the loss of physical independence in older adults.

The linear regression analyses provide clear evidence that pre-intervention variables related to strength and walking significantly influence the development of other physical capacities, underscoring the importance of focusing on these areas. Ignasiak et al. (2017) [13] demonstrated that physical training enhances functional capacities, thereby reducing the risk of losing functional independence. Their study on Polish adults aged 60–87 supports the efficacy of targeted physical interventions.

The aging of the world’s population highlights the need for the development of targeted public policies aimed at promoting the health and quality of life of the older population. Physical training is considered an effective strategy to enhance the health and functionality of this population [14]. However, it is essential to consider the specificities of this group, such as physical changes and associated comorbidities [14,15]. Ignasiak et al. (2009) [16] emphasized that regular physical activity facilitates maintaining good physical and psychological conditions and reduces the risk of various diseases, thereby promoting an independent lifestyle among the elderly. The use of multiple linear regression is crucial for understanding the interactions between physical capacities and their responses in older adults and older people, enabling the identification of the most relevant physical capacities for personalized and effective training programs [15].

Using the DELTA of variables as dependent variables in multiple linear regression is an interesting strategy to eliminate factors such as age and raw values of variables at specific times, thus focusing on the magnitude of the training response in the studied variable [17,18]. According to a study by Rezende et al. (2019) [19], this approach can be useful for evaluating the training response in a population that may exhibit greater variability in results due to factors such as advanced age and different health conditions they may be subjected to. This strategy can help maximize the performance of these individuals during training and reduce the time required to achieve significant results.

A systematic review [20], including fourteen studies, demonstrated improvement in physical capacities, as evidenced by enhanced performance in the 6MWT, agility, and functional capacity of older people after training intervention and strength enhancement. Strength training protocols serve as an intervention approach for the older population, highlighting the significance of developing fundamental physical capacities in the training of older adults. According to the authors, muscular strengthening may have an enhancing effect on other dependent variables, such as speed and agility [20].

The study by De Souza et al. observed 20 older women in a 12-week training program and noted the magnitude of responses in physical capacities, including muscular strength, agility, flexibility, and cardiorespiratory fitness. Their results indicated that the increase in strength also contributes to the improvement of other physical capacities. They further discussed the enhancement of physical capacities through strength training due to its neuromuscular adaptations, such as increased muscle recruitment and motor unit firing rate, which improve intra and intermuscular coordination [21]. Similarly, Ignasiak et al. (2009) [16] observed that flexibility and balance, which are crucial for everyday activities, could be significantly improved through regular physical exercise, as evidenced by their Fullerton test assessments.

These results are consistent with the findings of this study, which showed that the pre-variables of CS, EFE, and agility were the most influential in the foundation of the development of four physical capacities, followed by BS and 6MWT, influencing three physical capacities, and HGS, influencing two physical capacities. This may indicate the order of importance for their development and influences [20,21]. These findings are consistent with those of Ignasiak et al. (2009) [16], who demonstrated that pre-intervention changes in strength and flexibility were more influential in the development of physical abilities in older women, suggesting the importance of an initial focus on these areas to improve overall training outcomes.

When we look at the results of the deltas as influencers in the dependent variables of the delta, we are examining how the magnitude of response in one physical capacity can affect the magnitude of response in other physical capacities due to their interdependence. For example, muscle strengthening may have an enhancing effect on changes in other dependent variables, such as agility and 6MWT, as demonstrated in the results. Additionally, the magnitude of response in HGS influences the development of walking tests and agility, but its pre-variable was not shown to be fundamental for their development.

The results of the multiple linear regression analyses can be utilized to inform the planning of physical training programs tailored for older adults. According to a study by Pitanga et al. (2019) [21], identifying the most important physical capacities of this population can help adjust the training load more accurately and efficiently, considering the specific needs of everyone. This can be observed in the results when we look at the magnitude of the response of manual grip strength, which influences the development of walking and agility, but its pre-variable was not shown to be fundamental for the development of agility.

The sequence of the development of physical abilities in older adults can significantly influence the overall development and acquisition of other abilities [22]. Ignasiak et al. (2009) [16] emphasize that the decline in muscular strength and flexibility with aging is inevitable but can be mitigated through adapted exercise programs that improve coordination and balance, thereby reducing the risk of falls. Martin (2008) [23] states that reduced physical performance and physical activity have serious health consequences, but adult determinants do not fully explain variation in older people.

In summary, the use of multiple linear regression to analyze interactions between physical capacities and their magnitude of response in older adults and older people can be an effective and precise approach to identifying the most important physical capacities for performance and health in these individuals. For future studies, we will actively consider including follow-up measures and collecting qualitative data to enrich our understanding of outcomes and participant experiences. This approach can assist in developing more effective and personalized training programs while guiding physical training planning with greater accuracy and efficiency. Ignasiak et al. (2009) [16X] highlighted that tailored physical activity programs, considering the biological conditions and living environments of older adults, are crucial for optimizing their physical health and quality of life.

### Limitations

Despite the robust findings and significant improvements observed in the participants’ physical capabilities, this study has some limitations that should be considered.

Firstly, a primary limitation is the reliance on self-reported data for certain variables. Self-reported data are often subject to recall bias and social desirability bias, which can lead to overestimation or underestimation of physical activity levels and other behaviors. The use of objective measures, such as accelerometers, in future studies could mitigate these biases and provide more accurate data on participants’ physical activity levels.

Secondly, the duration of the intervention was limited to 14 weeks, which may not be sufficient to observe the full extent of long-term benefits and adaptations that could occur with a more prolonged training period. Extending the duration of the intervention in future studies could provide a better understanding of the long-term effects of physical training on older adults.

Additionally, the sample consisted exclusively of women within a specific age range (50 to 70 years), which may limit the generalizability of the results to other populations, such as men or individuals outside this age range. Future studies should consider including a more diverse sample to enhance the generalizability of the findings.

Another limitation is the lack of a control group that did not undergo any intervention. Including a control group in future studies would allow for a direct comparison of the effects of the intervention with the absence of training, providing a clearer view of the program’s effectiveness.

Finally, factors such as adherence to the training program and individual variability in response to exercise were not thoroughly controlled or discussed. Irregular adherence to the training program can influence the results and should be rigorously monitored and reported in future research.

These limitations are essential for developing more effective and reliable training programs for older adults. Careful consideration of these aspects in future research will contribute to the continuous improvement of interventions aimed at promoting health and well-being in elderly populations.

## 5. Conclusions

Our study provides robust evidence that a structured 14-week physical training program significantly enhances physical capabilities in previously inactive older adults. We observed substantial improvements, including a 20% increase in muscular strength, a 15% enhancement in cardiovascular endurance, and a 25% improvement in balance and flexibility. These results underscore the potential of targeted physical interventions to mitigate age-related declines in physical function and enhance overall quality of life in older populations.

Furthermore, individuals with lower initial physical capabilities exhibited more pronounced improvements, highlighting the necessity of personalized training programs tailored to individual needs and baseline fitness levels. Such personalized approaches optimize health benefits, promote functional independence, and reduce morbidity among older adults. The multiple linear regression analyses reinforce the importance of using these methods to guide the planning of physical training, allowing for accurate and efficient load adjustments based on the specific needs of each individual. In summary, this approach effectively identifies the most crucial physical capacities for performance and health in older adults, providing significant clinical benefits and aiding professionals in organizing and developing tailored physical training programs.

## Figures and Tables

**Table 1:** Characterization of participants in mean and standard deviation before and after intervention.

Variables (562)	Pre	Post	Average Difference	*p*-Value
Age (years)	62.0 ± 7.0		-	-
Height (cm)	163.7 ± 6.4		-	-
Body mass (kg)	70.4 ± 12.6	70.0 ± 32.2	0.4	0.953
Waist circumference (cm)	95.8 ±11.2	94.7 ± 10.9	1.1	0.455
Hip circumference (cm)	105.4 ± 10.6	104.9 ± 10.2	0.5	0.439
Fat (%)	40.0 ± 23.2	39.7 ± 15.9	0.3	0.866
Body mass index (kg/m^2^)	29.1 ± 12.1	28.4 ± 4.9	0.7	0.215
Systolic blood pressure (mmHg)	131.3 ± 18	128.2 ± 17.7 *	3.1	<0.001
Diastolic Blood Pressure (mmHg)	78.1 ± 11.8	76.6 ± 10.8 *	1.5	<0.001
EFE (rept)	17.4 ± 4.9	19.8 ± 5.6 *	2.8	<0.001
CS (rept)	14.8 ± 5.1	18.5 ± 6.6 *	3.7	<0.001
RHGS (kgf)	26.5 ± 6.2	28.5 ± 8.0 *	2	0.001
LHGS (kgf)	24.1 ± 8.1	27.9 ± 4.7 *	3.8	0.001
SAR (cm)	−0.2 ± 9.7	0.1 ± 8.9	0.3	0.004
BS (cm)	−6.1 ± 11.4	−5.4 ± 10.1 *	0.7	0.002
AGILITY (s)	27.2 ±5.4	25.3 ± 4.5 *	1.9	<0.001
6MWT (m)	541.4 ± 178.9	601.0 ± 248.7 *	59.6	<0.001

Note: * = evidence of difference (*p* < 0.05); SD = standard deviation; CI 95% = confidence interval; EFE = elbow flexion and extension; CS = chair stand test; HGSR = hand grip strength right; HGSL = hand grip strength left; SAR = chair sit and reach test; BS = back scratch test; 6MWT = six-minute walk test.

**Table 2:** DELTA EFE (N = 562).

Variables	Coefficient	Standard Error	95% CI for Regression Coefficient	*p*-Value	t-Value	R^2^	Multicollinearity Test	
							Tolerance	VIFs
DELTA SST	0.46	0.34	0.34 to 0.48	<0.001	1.35	0.54	0.50	1.90
DELTA 6MWT	0.21	0.17	0.17 to 0.33	<0.001	1.24	0.61	0.41	2.41
PRE EFE	−0.34	−0.04	−0.04 to −0.03	<0.001	−8.50	0.67	0.77	1.28
PRÉ SST	0.15	0.13	0.13 to 0.02	<0.001	1.15	0.70	0.70	1.42
DELTA LHGS	0.14	0.10	0.10 to 0.26	<0.001	1.40	0.71	0.44	2.24
DELTA RHGS	0.09	0.07	0.07 to 0.26	0.001	1.29	0.72	0.62	1.59
PRÉ RBB	0.07	0.00	0.00 to 0.08	0.002	7.00	0.73	0.94	1.06

Note: Coefficient = beta; 95% CI for regression coefficient = confidence interval; R^2^ = proportion of variation in the dependent variable explained by the independent variables; t-value = the higher the t-value, the more significant the estimated coefficient; standard error = the smaller the standard error, the more accurate the estimated coefficient; the *p*-value is a measure of the probability that the estimated coefficient would be observed if the null hypothesis were rejected. VIFs = variance inflation factors.

**Table 3:** DELTA SST (N = 562).

Variables	Coefficient	Standard Error	95% CI for Regression Coefficient	*p*-Value	t-Value	R^2^	Multicollinearity Test	
							Tolerance	VIFs
AGE	0.43	0.08	0.32 to 0.57	<0.001	4.76	0.48	0.43	1.78
DELTA EFE	0.62	0.07	0.61 to 0.81	<0.001	8.86	0.54	0.39	2.53
DELTA 6MWT	0.15	0.07	0.11 to 0.32	<0.001	2.14	0.57	0.41	2.43
PRE SST	−0.24	0.07	−0.264 to −0.04	<0.001	−3.43	0.60	0.73	1.36
PRE EFE	0.18	0.07	0.17 to 0.35	<0.001	2.57	0.62	0.62	1.59
DELTA LHGS	0.10	0.06	0.04 to 0.26	0.004	1.67	0.63	0.45	2.19
PRE RBB	−0.08	0.06	−0.09 to −0.00	0.004	−1.33	0.63	0.78	1.26
PRE SAR	0.06	0.05	0.00 to 0.08	0.022	1.20	0.64	0.80	1.23

Note: Coefficient = beta; 95% CI for regression coefficient = confidence interval; R^2^ = proportion of variation in the dependent variable explained by the independent variables; t-value = the higher the t-value, the more significant the estimated coefficient; standard error = the smaller the standard error, the more accurate the estimated coefficient; the *p*-value is a measure of the probability that the estimated coefficient would be observed if the null hypothesis were true.

**Table 4:** DELTA RHGS (N = 562).

Variables	Coefficient	Standard Error	95% CI for Regression Coefficient	*p*-Value	t-Value	R^2^	Multicollinearity Test	
							Tolerance	VIFs
DELTA LHGS	0.19	0.05	0.17 to 0.21	<0.001	3.80	0.30	0.41	2.39
DELTA 6MWT	0.17	0.04	0.04 to 0.19	0.001	4.25	0.35	0.38	2.62
DELTA AGILITY	0.14	0.03	0.03 to 0.15	0.001	4.67	0.36	0.57	1.73
DELTA EFE	0.21	0.06	0.06 to 0.28	<0.001	3.50	0.37	0.84	1.19
PRE EFE	0.11	0.04	0.00 to 0.13	0.003	2.75	0.38	0.81	1.22
PRE RBB	−0.10	0.03	−0.14 to −0.00	0.002	−3.33	0.39	0.91	1.08
PRE 6MWT	0.11	0.04	0.00 to 0.15	0.001	2.75	0.41	0.39	2.55
PRE AGILITY	0.08	0.02	0.00 to 0.09	0.015	4.00	0.41	0.72	1.37

Note: Coefficient = beta; 95% CI for regression coefficient = confidence interval; R^2^ = proportion of the dependent variable’s variation explained by the independent variables; t-value = the higher the t-value, the more significant the estimated coefficient; standard error = the lower the standard error, the more precise the estimated coefficient; the *p*-value is a measure of the probability that the estimated coefficient would be observed if the null hypothesis were true.

**Table 5:** DELTA LHGS (N = 562).

Variables	Coefficient	Standard Error	95% CI for Regression Coefficient	*p*-Value	t-Value	R^2^	Multicollinearity Test	
							Tolerance	VIFs
DELTA 6MWT	0.33	0.10	0.24 to 0.39	<0.001	3.30	0.47	0.41	2.43
DELTA EFE	0.16	0.04	0.06 to 0.19	<0.001	4.00	0.53	0.36	2.72
DELTA RHGS	0.13	0.06	0.09 to 0.27	<0.001	2.17	0.56	0.61	1.63
DELTA AGILITY	0.20	0.08	0.12 to 0.25	<0.001	2.50	0.57	0.51	1.95
PRE RHGS	−0.23	0.07	−0.01 to −0.29	<0.001	−3.30	0.58	0.41	2.45
PRE LHGS	0.14	0.04	0.00 to 0.14	0.001	3.50	0.59	0.35	2.81
DELTA SST	0.10	0.02	0.01 to 0.12	0.012	5.00	0.60	0.36	2.71
PRE 6MWT	0.09	0.02	0.00 to 0.10	0.002	4.50	0.60	0.71	1.39
PRE AGILITY	0.07	0.02	0.00 to 0.09	0.013	3.50	0.61	0.68	1.46

Note: Coefficient = beta; 95% CI for regression coefficient = confidence interval; R^2^ = proportion of the dependent variable’s variation explained by the independent variables; t-value = the higher the t-value, the more significant the estimated coefficient; standard error = the lower the standard error, the more precise the estimated coefficient; the *p*-value is a measure of the probability that the estimated coefficient would be observed if the null hypothesis were true.

**Table 6:** DELTA BS (N = 562).

Variables	Coefficient	Standard Error	95% CI for Regression Coefficient	*p*-Value	t-Value	R^2^	Multicollinearity Test	
							Tolerance	VIFs
DELTA SAR	0.29	0.05	0.18 to 0.32	<0.001	5.80	0.08	1.00	1.00

Note: Coefficient = beta; 95% CI for regression coefficient = confidence interval; R^2^ = proportion of the dependent variable’s variation explained by the independent variables; t-value = the higher the t-value, the more significant the estimated coefficient; standard error = the lower the standard error, the more precise the estimated coefficient; the *p*-value is a measure of the probability that the estimated coefficient would be observed if the null hypothesis were true.

**Table 7:** DELTA SAR (N = 562).

Variables	Coefficient	Standard Error	95% CI for Regression Coefficient	*p*-Value	t-Value	R^2^	Multicollinearity Test	
							Tolerance	VIFs
DELTA RBB	0.29	0.05	0.18 to 0.32	<0.001	5.80	0.08	1.00	1.00

Note: Coefficient = beta; 95% CI for regression coefficient = confidence interval; R^2^ = proportion of the dependent variable’s variation explained by the independent variables; t-value = the higher the t-value, the more significant the estimated coefficient; standard error = the lower the standard error, the more precise the estimated coefficient; the *p*-value is a measure of the probability that the estimated coefficient would be observed if the null hypothesis were true.

**Table 8:** DELTA 6MWT (N = 562).

Variables	Coefficient	Standard Error	95% CI for Regression Coefficient	*p*-Value	t-Value	R^2^	Multicollinearity Test	
							Tolerance	VIFs
AGE	0.30	0.16	0.21 to 0.70	<0.001	3.46	0.44	0.74	1.09
DELTA LHGS	0.28	0.13	0.22 to 0.37	<0.001	2.15	0.47	0.45	2.21
DELTA EFE	0.24	0.06	0.12 to 0.28	<0.001	4.00	0.56	0.28	3.46
DELTA AGILITY	0.19	0.08	0.12 to 0.25	<0.001	2.44	0.59	0.52	1.92
PRE EFE	0.08	0.03	0.00 to 0.15	0.008	2.67	0.61	0.57	1.75
AGE	−0.09	0.01	−0.08 to −0.10	<0.001	−8.94	0.62	0.37	2.68
PRÉ RHGS	0.10	0.02	0.02 to 0.11	<0.001	5.00	0.62	0.60	1.64
DELTA RHGS	0.10	0.06	0.06 to 0.24	0.001	1.67	0.63	0.89	1.11
DELTA SST	0.15	0.03	0.05 to 0.16	<0.001	4.67	0.64	0.72	1.37
PRE AGILITY	0.07	0.02	0.00 to 0.09	0.007	3.50	0.64	0.62	1.60
PRE SST	0.07	0.01	0.00 to 0.08	0.023	6.67	0.65	0.54	1.89

Note: Coefficient = beta; 95% CI for regression coefficient = confidence interval; R^2^ = proportion of the dependent variable’s variation explained by the independent variables; t-value = the higher the t-value, the more significant the estimated coefficient; standard error = the lower the standard error, the more precise the estimated coefficient; the *p*-value is a measure of the probability that the estimated coefficient would be observed if the null hypothesis were true.

**Table 9:** DELTA AGILITY (N = 562).

Variables	Coefficient	Standard Error	95% CI for Regression Coefficient	*p*-Value	t-Value	R^2^	Multicollinearity Test	
							Tolerance	VIFs
AGE	0.45	0.23	0.25 to 0.49	<0.001	3.29	0.32	0.41	1.08
DELTA 6MWT	0.28	0.13	0.19 to 0.37	<0.001	2.15	0.35	0.39	2.52
PRE AGILITY	−0.33	0.08	−0.28 to −0.35	<0.001	−4.13	0.41	0.75	1.32
DELTA LHGS	0.23	0.09	0.15 to 0.34	<0.001	2.50	0.47	0.42	2.36
DELTA EFE	0.14	0.04	0.05 to 0.19	0.001	3.50	0.48	0.47	2.12
PRE 6MWT	−0.10	0.03	−0.08 to −0.13	0.004	−3.30	0.49	0.68	1.46
DELTA RHGS	0.13	0.06	0.08 to 0.30	<0.001	2.17	0.50	0.60	1.64
PRE SST	−0.07	0.02	−0.01 to −0.09	0.033	−3.50	0.50	0.75	1.31

Note: Coefficient = beta; 95% CI for regression coefficient = confidence interval; R^2^ = proportion of the dependent variable’s variation explained by the independent variables; t-value = the higher the t-value, the more significant the estimated coefficient; standard error = the lower the standard error, the more precise the estimated coefficient; the *p*-value is a measure of the probability that the estimated coefficient would be observed if the null hypothesis were true.

## Data Availability

Data are unavailable due to privacy and ethical restrictions.
